# Extracellular NAD^+^ response to post-hepatectomy liver failure: bridging preclinical and clinical findings

**DOI:** 10.1038/s42003-024-06661-0

**Published:** 2024-08-14

**Authors:** Can Kamali, Philipp Brunnbauer, Kaan Kamali, Al-Hussein Ahmed Saqr, Alexander Arnold, Gulcin Harman Kamali, Julia Babigian, Eriselda Keshi, Raphael Mohr, Matthäus Felsenstein, Simon Moosburner, Karl-Herbert Hillebrandt, Jasmin Bartels, Igor Maximilian Sauer, Frank Tacke, Moritz Schmelzle, Johann Pratschke, Felix Krenzien

**Affiliations:** 1 Charité – Universitätsmedizin, corporate member of Freie Universität Berlin, Humboldt-Universität zu Berlin, Department of Surgery - Campus Charité Mitte and Campus Virchow-Klinikum, Augustenburger Platz 1, 13353 Berlin, Germany; 2 Charité – Universitätsmedizin, corporate member of Freie Universität Berlin, Humboldt-Universität zu Berlin, Institute of Pathology, Charitéplatz 1, 10117 Berlin, Germany; 3 University of Health Sciences, Prof. Dr. Cemil Taşçıoğlu City Hospital, Department of Pathology, Istanbul, Turkey; 4 Berlin Institute of Health at Charité – Universitätsmedizin Berlin, BIH Academy, Clinician Scientist Program, Charitéplatz 1, 10117 Berlin, Germany; 5 Charité – Universitätsmedizin, corporate member of Freie Universität Berlin, Humboldt-Universität zu Berlin, Department of Hepatology and Gastroenterology - Campus Charité Mitte and Campus Virchow-Klinikum, Augustenburger Platz 1, 13353 Berlin, Germany; 6 Hannover Medical School, Department of General, Visceral and Transplant Surgery, Carl-Neuberg-Straße 1, 30625 Hannover, Germany

**Keywords:** Liver cirrhosis, Translational research

## Abstract

Liver fibrosis progressing to cirrhosis is a major risk factor for liver cancer, impacting surgical treatment and survival. Our study focuses on the role of extracellular nicotinamide adenine dinucleotide (eNAD^+^) in liver fibrosis, analyzing liver disease patients undergoing surgery. Additionally, we explore NAD^+^’s therapeutic potential in a mouse model of extended liver resection and in vitro using 3D hepatocyte spheroids. eNAD^+^ correlated with aspartate transaminase (AST) and bilirubin after liver resection (AST: *r* = 0.2828, *p* = 0.0087; Bilirubin: *r* = 0.2584, *p* = 0.0176). Concordantly, post-hepatectomy liver failure (PHLF) was associated with higher eNAD^+^ peaks (*n* = 10; *p* = 0.0063). Post-operative eNAD^+^ levels decreased significantly (*p* < 0.05), but in advanced stages of liver fibrosis or cirrhosis, this decline not only diminished but actually showed a trend towards an increase. The expression of NAD^+^ biosynthesis rate-limiting enzymes, nicotinamide phosphoribosyltransferase (NAMPT) and nicotinamide mononucleotide adenylyltransferase 3 (NMNAT3), were upregulated significantly in the liver tissue of patients with higher liver fibrosis stages (*p* < 0.0001). Finally, the administration of NAD^+^ in a 3D hepatocyte spheroid model rescued hepatocytes from TNFalpha-induced cell death and improved viability (*p* < 0.0001). In a mouse model of extended liver resection, NAD^+^ treatment significantly improved survival (*p* = 0.0158) and liver regeneration (*p* = 0.0186). Our findings reveal that eNAD^+^ was upregulated in PHLF, and rate-limiting enzymes of NAD^+^ biosynthesis demonstrated higher expressions under liver fibrosis. Further, eNAD^+^ administration improved survival after extended liver resection in mice and enhanced hepatocyte viability in vitro. These insights may offer a potential target for future therapies.

## Abstract (web summary)

An analysis on liver disease patients suggests that extracellular Nicotinamide adenine dinucleotide (eNAD ^+ ^) correlates with liver fibrosis and post-hepatectomy liver failure, with NAD^+^ administration improving survival and liver regeneration in mice and hepatocyte viability in vitro.

## Introduction

The intricate progression of liver fibrosis leading to cirrhosis continues to be a worldwide concern and serves as the primary determinant for the onset of liver cancer^1,2^. Limited by this is liver resection, which remains the cornerstone of oncologic therapy of liver cancer and is of paramount importance for survival^3,4^. The significance of cirrhosis is highlighted by the prevalence of hepatocellular carcinoma, which ranks as the second cause of cancer-related death worldwide^5^. In modern clinical practice, the implementation of multimodal oncological concepts often involves an extended liver resection or even a two-stage hepatectomy including a clearance of the left liver followed by a hypertrophy period and subsequent resection of the right liver. However, the success of these procedures relies significantly on the liver’s ample function and regenerative capacity^3,6,7^.

Nicotinamide adenine dinucleotide (NAD^+^) and its regenerative properties have been recognized for their involvement in many intricate cellular functions; the molecule serves as an electron transporter in redox reactions, plays central roles in both glucose breakdown and synthesis, contributes to DNA repair mechanisms, and acts as a protective agent against reactive oxygen species^8^. While there is evidence^9^ suggesting that overall NAD^+^ concentrations may not uniformly decrease with age across the entire system as described before^10,11^, studies have rather demonstrated a decline in specific tissues and cells^12–14^. NAD^+^ can be generated in three different ways: de-novo synthesis, the Preis-Handler pathway, and the salvage pathway. Interestingly, NAD^+^ can be found in intracellular and in extracellular compartments. While the intracellular concentration of NAD^+^ is around 500 times higher than that of plasma or other extracellular compartments^15^, research has mostly focused on intracellular NAD^+^ ^16^. A flux quantification via isotope-tracers revealed in vivo that the liver appeared to be the only organ comprising all the necessary enzymes for the de-novo synthesis pathway of NAD^+^ ^17^. In addition, the liver was found not only to consume the most tryptophan for NAD^+^ biosynthesis^18^, but was also capable of excreting nicotinic acid (NA) and supply other organs with NAD^+^ precursors via haemocirculation^17^.

In contrast to the exhaustive research on intracellular NAD^+^ (iNAD^+^) there is little to no data available on extracellular NAD^+^ ^19,20^ (eNAD^+^) and its role within the comprehensive human physiological framework. The quantification of this metabolite, exhibiting concentrations in the high nanomolar range in plasma, requires a particularly robust, sensitive and reproducible methodology^21^. Currently used quantification methods, based on liquid chromatography and mass spectrometry, are highly complex have limited reproducibility and consequently prove inadequate for conducting high-throughput clinical screenings^22^. In a previous publication, we described a novel two-step enzymatic assay for detecting eNAD^+^ in the nanomolar range in human heparinized plasma with the help of albumin modified simulated body fluids^23^.

To address the lack of evidence in eNAD^+^ research, we present data derived from a cohort study involving patients who underwent liver resection. In addition, we present data from cell culture and animal studies showing that NAD^+^ administration improved hepatic viability in vitro and survival after liver resection in vivo. Our objective is to offer insights into the ramifications of altered eNAD^+^ levels in patients with liver disease. This investigation holds promise as a prospective avenue for future therapeutic targets.

## Results

### Liver fibrosis, extent of liver resection and eNAD^+^

**Fig. 1:**
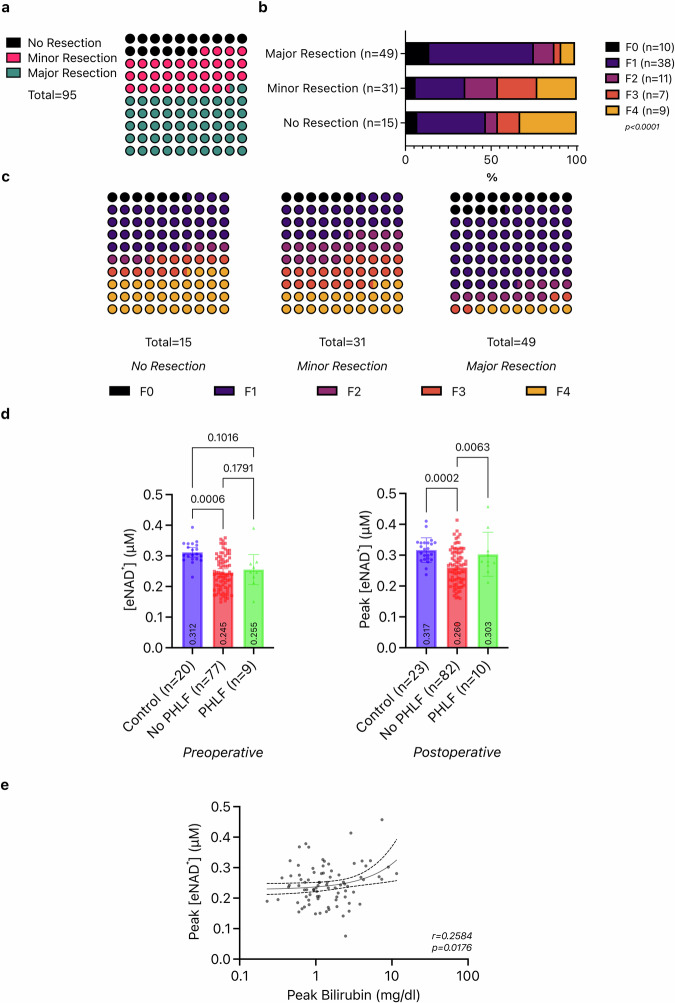
Clinical parameters, liver fibrosis and eNAD^+^. **a** Distribution of the patients classified according to the extent of the liver resection. **b**, **c** Patients grouped according the extent of the resection and their liver fibrosis stage, compared by Chi-squared test. **d** Patients undergoing liver resection are divided into subgroups based on whether they developed PHLF and are compared with two-way ANOVA. Bars represent mean levels with scatter plot for individual values, error lines stand for standard deviation. **e** Peak eNAD^+^ levels are correlated to peak bilirubin levels by Pearson correlation (base-2 logarithmic scale).

Initially, we reviewed the distribution of major and minor liver resections of the present cohort (Table 1): more than half (51.6%) of all liver resection patients underwent a major liver resection, which included right, left and extended hepatectomies, the rest of the hepatectomies were minor resections (less than 3 segments). (Fig. 1a) Next, we explored the relationship between liver fibrosis and the extent of liver resection. Major resections were predominantly performed on livers with lower fibrosis stages, whereas smaller resections were carried out more frequently with increasing fibrosis (Fig. 1b, c, *p* < 0.0001).

**Table 1:** Demographic characterization of the patient cohorts

Characteristics	Liver Resection		Control
**Number of patients**	95		24
**Age on the day of surgery (Years)**	M = 62.8 (26–86)		M = 59 (29–81)
**Sex**			
: Female	36.8% (35)		41.7% (10)
: Male	63.2% (60)		58.3% (14)
**Body Mass Index (kg/m²)**	M = 26.4 (17.3–40.3)		M = 28.3 (23.1–39.9)
Underweight (BMI = < 18.5 kg/m^2^)	3.2% (3)		0% (0)
Normal (BMI = 18.5–< 25 kg/m^2^)	26.3% (25)		16.0% (4)
Overweight (BMI = 25–< 30 kg/m^2^)	41.1% (39)		44.0% (11)
Grade 1 Obesity (BMI = 30–< 35 kg/m^2^)	13.7% (13)		20.0% (5)
Grade 2 Obesity (BMI = 35–< 40 kg/m^2^)	1.1% (1)		4% (1)
Grade 3 Obesity (BMI = > 40 kg/m^2^)	1.1% (1)		0% (0)
unidentified	13.7% (13)		16.0% (4)
**Type of resection (Brisbane Criteria)**			-
No resection	15.8% (15)		-
Minor Resection	32.6% (31)		-
Major Resection	51.6% (49)		-
**Stage of fibrosis (Desmet)**			-
No fibrosis	10.5% (10)		-
Stage 1	47.3% (45)		-
Stage 2	13.6% (13)		-
Stage 3	11.6% (11)		-
Stage 4	16.8% (16)		-
**Grade of steatosis (%)**	*M* = 20,8 (0–70)		-
**Entity**			-
: Living Donor Transplant	3.2% (3)		-
: Hepatocellular Carcinoma (HCC)	26.3% (25)		-
: Intrahepatic Cholangiocellular Carcinoma (iCC)	12.6% (12)		-
: Extrahepatic Cholangiocellular Carcinoma (Klatskin Tumor - eCC)	11.6% (11)		
Metastasis of …			
: …Colorectal Cancer (CRLM)	48.4% (46)		-
: …Adrenocortical Cancer	1.1% (1)	Others % (31)	-
: …Anal Cancer	1.1% (1)		-
: …Breast Cancer	1.1% (1)		-
: …Gastrointestinal Stromal Tumor (GIST)	2.1% (2)		-
: …Granulosa Cell Tumor	1.1% (1)		-
: …Leiomyosarcoma	1.1% (1)		-
: …Liposarcoma	1.1% (1)		-
: …Neuroendocrine Tumor (NET)	1.1% (1)		-
: …Neuroendocrine Cancer (NEC)	3.2% (3)		-
: …Non-Small Cell Lung Cancer (NSCLC)	1.1% (1)		-
: …Stomach Cancer	1.1% (1)		-
: Caroli Syndrome	3.2% (3)		-
: Cavernous Hemangioma	2.1% (2)		-
: Hemangiosarcoma	1.1% (1)		-
: Focal Nodular Hyperplasia (FNH)	1.1% (1)		-
: Alcohol-related Liver Disease (ALD)	1.1% (1)		-
: Hepatitis C Virus (HCV)	8.4% (8)		-
: Primary Sclerosing Cholangitis (PSC)	1.1% (1)		-
Pre-operative LiMAX*			-
**: Normal Liver Function (>** **315** **µg/kg/h)**	55,8% (53)		-
**: Impaired Liver Function (<** **315** **µg/kg/h)**	26,3% (25)		-
**: Undefined**	17,9% (17)		-
**Posthepatectomy liver failure (ISGLS)**	10,5% (10)		-
**: Grade A**	20% (2)		-
**: Grade B**	20% (2)		-
**: Grade C**	60% (6)		-

### Post-hepatectomy liver failure and liver fibrosis

In the cohort under study, 10 out of 92 patients (10.8%) were diagnosed with Post-hepatectomy liver failure (PHLF) according to the ISGLS grading system^24^. Among these, 60% were classified as the most severe Grade C PHLF, 20% as Grade B and 20% as Grade A. Levels of eNAD^+^ in both groups (with and without PHLF) were compared before and after surgery against each other and control patients. Pre-operatively, liver resection patients with or without PHLF exhibited statistically significantly lower eNAD^+^ levels (no PHLF: *n* = 77, *M* = 0.245 ± 0.057 µM; *p* = 0.0006; PHLF: *n* = 10, *M* = 0.255 ± 0.064 µM, *p* = 0.1791) than the control group (*n* = 20; *M* = 0.312 ± 0.034 µM). There was no difference between patients with or without PHLF pre-operatively (Pre-Op) (*p* = 0.1791, Fig. 1d). Following surgery, patients with PHLF exhibited significantly higher levels of eNAD^+^ (*M* = 0.303 ± 0.071 µM) compared to patients without liver dysfunction (*M* = 0.260 ± 0.061 µM; *p* = 0.0063), while there was no difference to the control group anymore (*M* = 0.317 ± 0.399 µM; *p* = 0.9950). Likewise, peak bilirubin levels increased significantly with increasing peak eNAD^+^ levels in a low positive correlation (*r* = 0.2584, *p* = 0.0176) (Fig. 1e).

**Fig. 2:**
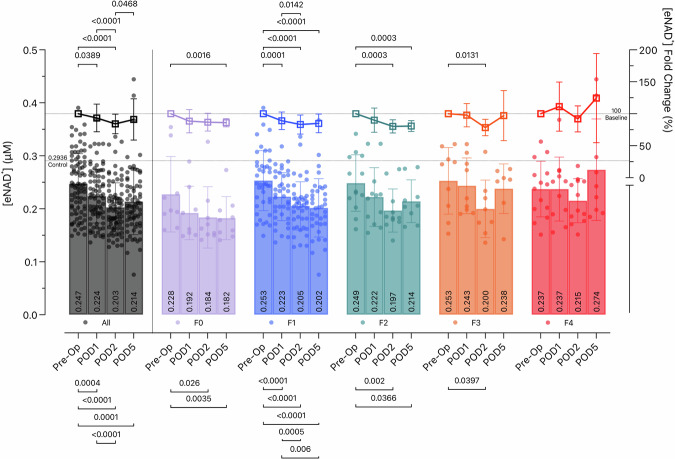
eNAD^+^ dynamics after liver resection and liver fibrosis. Absolute eNAD^+^ values (µM) shown in filled circles, left y-axis. eNAD^+^ fold changes (%) from the Pre-Op baseline levels shown in squares, right y-axis. Bars represent mean levels with scatter plot for individual values, error lines stand for standard deviation. Black stands for the main group of liver resection patients. Fibrosis stages are demonstrated in different colors, purple for no fibrosis (F0), blue for F1, green for F2, orange for F3 and red for cirrhosis (F4).

We explored the dynamics of eNAD^+^ concentrations in liver resection patients, focusing on the impact of liver fibrosis on these changes (Fig. 2). Pre-operative mean eNAD^+^ levels in liver resection patients were at 0.247 µM (± 0.057). Post-operatively, these patients showed significant decreases in eNAD^+^ levels from Pre-Op to POD1 (*M* = 0.224 ± 0.050 µM, *p* = 0.004), POD2 (*M* = 0.203 ± 0.046 µM, *p* < 0.0001) and to POD5 (*M* = 0.214 ± 0.062 µM, *p* = 0.0001), as well as from POD1 to POD2 (*p* < 0.0001). To investigate the influence of liver fibrosis on these dynamics, we observed that the post-operative eNAD^+^ decrease was most pronounced in patients with lower fibrosis stages (F0 to F2): In the F0 group from Pre-Op (*M* = 0.228 ± 0.071 µM) to POD2 (*M* = 0.184 ± 0.058 µM, *p* = 0.026) and to POD5 (*M* = 0.182 ± 0.041 µM, *p* = 0.0035). In the F1 group from Pre-Op (*M* = 0.253 ± 0.056 µM) to POD1 (*M* = 0.223 ± 0.452 µM, *p* < 0.0001), to POD2 (*M* = 0.205 ± 0.046 µM, *p* < 0.0001) and to POD5 (*M* = 0.202 ± 0.055 µM, *p* < 0.0001), as well as from POD1 to POD2 (*p* = 0.0005) and to POD5 (*p* = 0.0006). In the F2 group from Pre-Op (*M* = 0.249 ± 0.053 µM) to POD2 (*M* = 0.197 ± 0.041 µM, *p* = 0.002) and to POD5 (*M* = 0.214 ± 0.040 µM, *p* = 0.0366). Patients with F3 fibrosis demonstrated a similar but less substantial decline from Pre-OP (*M* = 0.253 ± 0.063 µM) to POD5 (*M* = 0.238 ± 0.047 µM, *p* = 0.0397), while those with F4 fibrosis exhibited no significant decrease. Instead, a non-significant trend towards an increase in eNAD^+^ levels was noted in this group. Additionally, we analyzed the fold change in eNAD^+^ values relative to each patient’s baseline (Pre-Op) concentration. This analysis further supported our findings, reinforcing the observed patterns in absolute eNAD^+^ values and highlighting the differential impact of fibrosis stage on eNAD^+^ dynamics following liver resection.

**Fig. 3:**
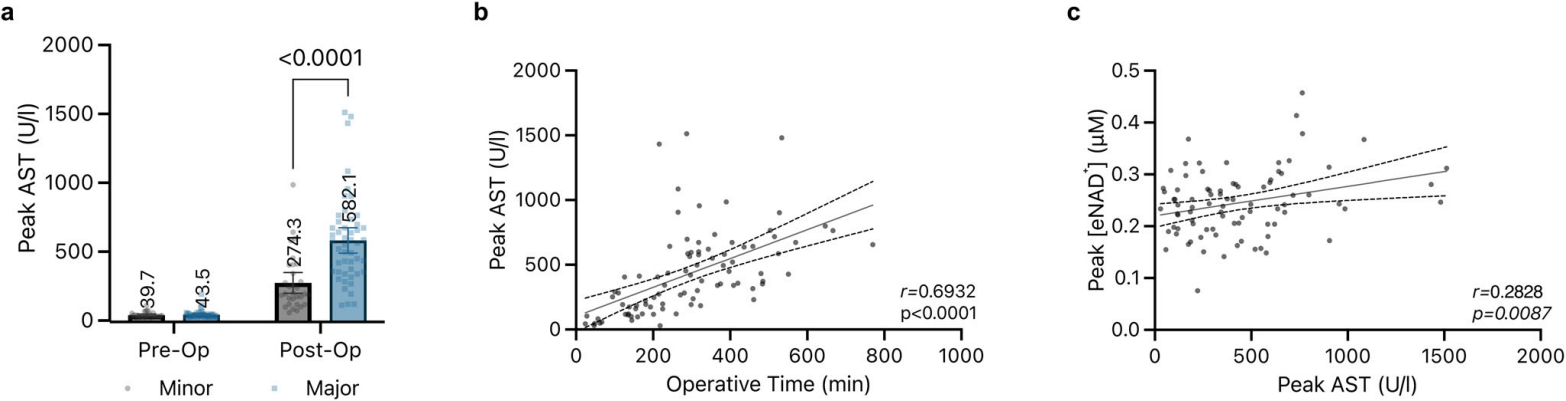
Extent of liver resection, AST and eNAD^+^. **a** Peak AST values of major and minor liver resections compared (two-way ANOVA with Šidák correction), both pre- and post-operatively. **b** Peak AST levels are correlated to operative time by Spearman correlation (**c**) Peak eNAD^+^ levels are correlated to peak AST levels by Pearson correlation.

Moreover, we analyzed the relationship between the release of aspartate aminotransferase (AST) and eNAD^+^ levels following liver resection. Notably, patients undergoing major resections exhibited significantly higher peaks AST (*M* = 582.1 ± 320.0 U/l, *p* < 0.0001) compared to those who had minor resections (*M* = 274.3 ± 206.0 U/l) (Fig. 3a). Further analysis revealed that post-operative AST levels increased in proportion to the length of the operative time (Pearson correlation, *n* = 89, *r* = 0.5442, *p* < 0.0001; Fig. 3b). Additionally, there was a significant positive correlation between post-operative peak AST levels and peak eNAD^+^ levels (Pearson correlation, *n* = 86, *r* = 0.2828, *p* = 0.0087) (Fig. 3c).

### NAMPT/NMNAT3 and liver fibrosis

**Fig. 4:**
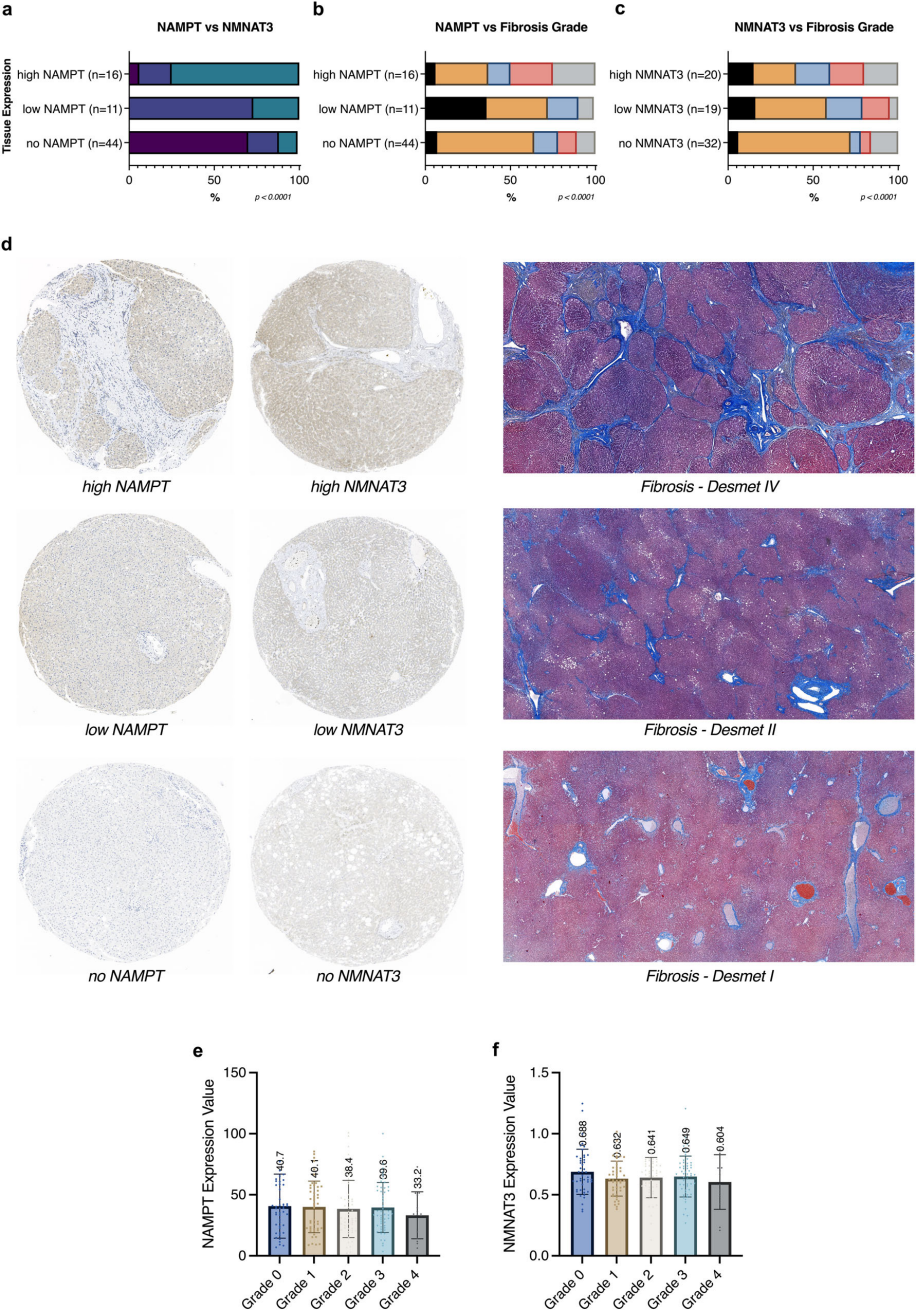
NAMPT/NMNAT3 and liver fibrosis. **a** The rate-limiting enzymes of the NAD^+^ biosynthesis, NAMPT and NMNAT, shown in association to each other and **b**, **c** to the stage of fibrosis of their corresponding liver tissue, analysed in contingency tables for their frequency distribution via Chi-squared test. **d** Representative immunohistochemical and histopathological slides of the nucleic enzyme expressions (NAMPT & NMNAT3, brown), cytoplasma counterstained with hematoxylin (blue) and the liver fibrosis in the corresponding tissue. **e** Gene expression values of patients with non-alcoholic fatty liver disease (*n* = 206) and control participants (*n* = 10) grouped according to their liver fibrosis stage^26^ NAMPT Expression and **f** NMNAT3 expression analysed with Kruskal-Wallis test. Bars represent mean levels with scatter plot, error lines represent standard deviation.

We measured the expression levels of nicotinamide phosphoribosyltransferase (NAMPT) and nicotinamide phosphoribosyltransferase (NMNAT3), the key enzymes in the NAD^+^ salvage pathway, in the liver resectates. The expression levels of both enzymes were highly concordant with each other: high expression of NAMPT was associated with high expression of NMNAT3 (Fig. 4a) and high expression of both of these enzymes was also observed in patients with high fibrosis stages (Fig. 4b, c). The corresponding immunohistochemical and histopathological slides used for the aforementioned analysis are shown representatively in Fig. 4d. Next, our aim was to determine whether the higher expressions of rate-limiting enzymes were attributable to higher RNA expression levels as well. Therefore, we utilised a separate external dataset^25^ (*n* = 216) to analyse the gene expressions of these enzymes on a RNA transcriptome level, consisting of 206 MAFLD (formerly known as *NAFLD)* patients with varying fibrosis stages and 10 healthy obese patients. After grouping the dataset based on liver fibrosis stages in the Gene Expression Omnibus, we compared expression values of NMNAT3 and NAMPT. Although there was a slight trend in expression for both enzymes as the fibrosis stage increased, the observed changes did not reach statistical significance (Fig. 4e, f).

### Treatment of 3D liver organoids with eNAD^+^

**Fig. 5:**
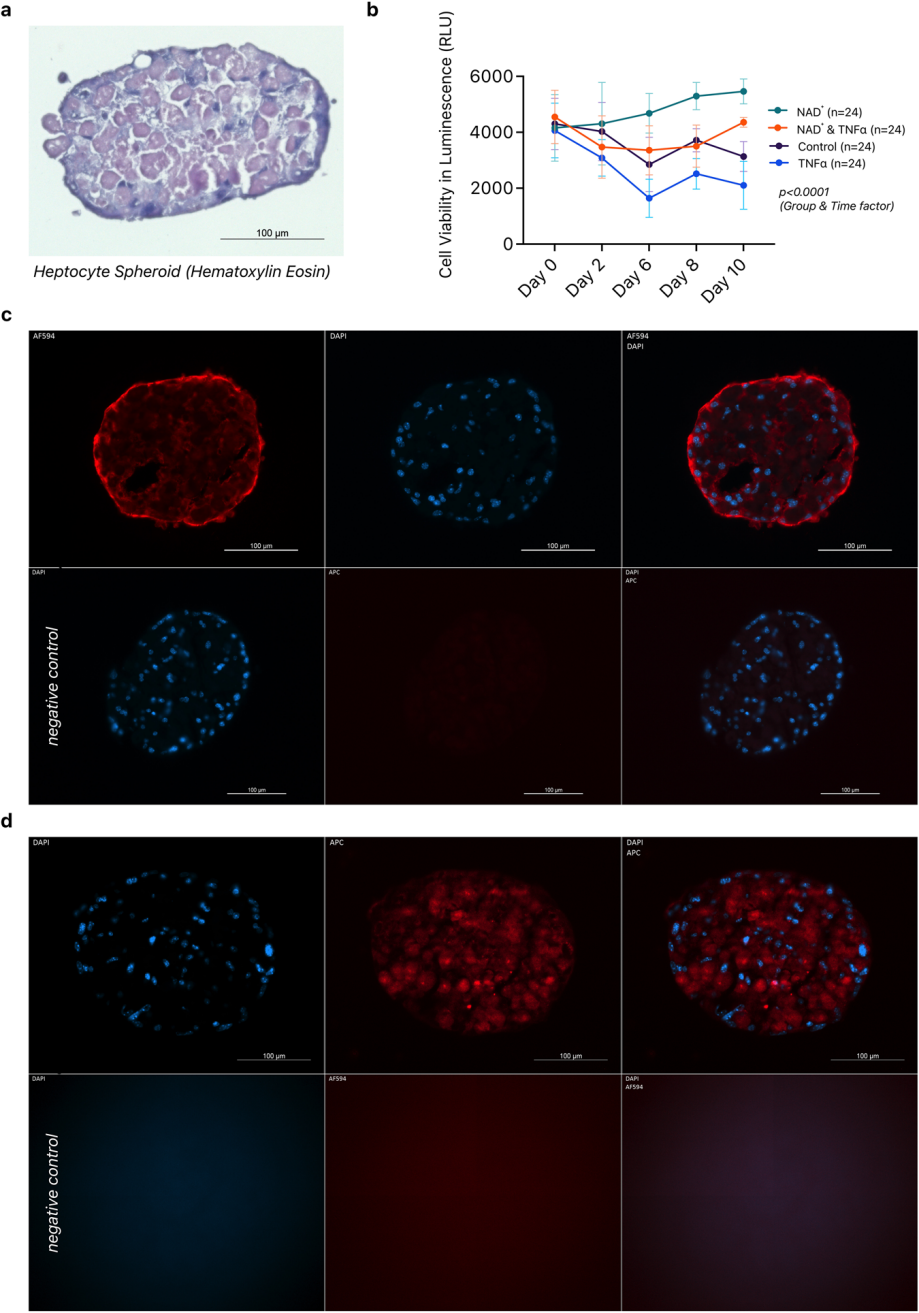
Treatment of 3D Liver Organoids with NAD^+^. **a** A formation of hepatocyte spheroids fixated in formaldehyde and stained with hematoxylin and eosin. **b** Cell viability of these spheroids, divided into 4 treatment groups (NAD^+^ treatment, NAD^+^ & *TNFalpha* treatment, control and *TNFalpha* treatment) assessed on pre-specified timepoints via luminescence, statistically analysed with two-way ANOVA. Error lines represent standard deviation. **c** Cell specificity and formation demonstrated with *Alexa594 conjugated Albumin* and **d** *APC coupled Cytokeratin 18* immunofluorescence staining with both DAPI counterstaining, and their corresponding negative controls.

We observed decreased eNAD^+^ levels post-operatively but increased levels in patients with fibrosis and PHLF, prompting us to test NAD^+^ treatment’s effect on liver viability in a pre-clinical setting. A cultivated hepatocyte spheroid is representatively shown in Fig. 5a after formaldehyde fixation and H&E staining.

The control group, with no treatments, exhibits a gradual decrease in cell viability, with levels declining to around 3000 RLU (*M* = 3133 ± 531.9 RLU) by Day 10. The hepatocytes treated first with *TNFalpha* and then NAD^+^ exhibited an increasing curve in their viability higher than both the *TNFalpha* treatment and control group. Cell viability here initially decreases by Day 2 but then stabilizes around 3500 RLU (*M* = 3474 ± 1114 RLU) from Day 6 to Day 10. The NAD^+^ treatment mitigated the effect of *TNFalpha* application and increased cell viability of the hepatocytes. The group treated only with *TNFalpha* demonstrated the lowest viability among all the other groups during the entire experiment, with levels declining to around 2000 RLU (*M* = 2103 ± 856.7 RLU) by Day 10. Hepatocytes treated with NAD^+^ alone show a consistent increase in cell viability over time, reaching approximately 5500 RLU (*M* = 5462 ± 444.0 RLU) by Day 10 and result in higher cell viability compared to other treatments, particularly significant on Day 6 (Control vs. NAD^+^ Treatment: *MD* = −1885 RLU, *p* < 0.0001, *TNFalpha* Treatment vs. NAD^+^ Treatment: *MD* = −3094 RLU, *p* < 0.0001 and NAD^+^ Treatment vs. NAD^+^ and *TNFalpha* Treatment: *MD* = 1380 RLU, *p* = 0.0093). The mixed-effects model analysis indicates highly significant effects of time, treatment groups, and their interaction on hepatocyte viability (*p* < 0.0001 for all). The significant interaction between time and treatment groups (*p* < 0.0001) suggests that the effect of treatments on cell viability changes over time. (Fig. 5b).

Hepatocyte formation and specificity of the cell type were confirmed via immunofluorescence staining utilising *APC-coupled Albumin* (Fig. 5c) and *Alexa594-coupled Cytokeratin 18* (Fig. 5d) antibodies with DAPI counterstaining.

### Extended liver resection in mice under NAD^+^ treatment

**Fig. 6:**
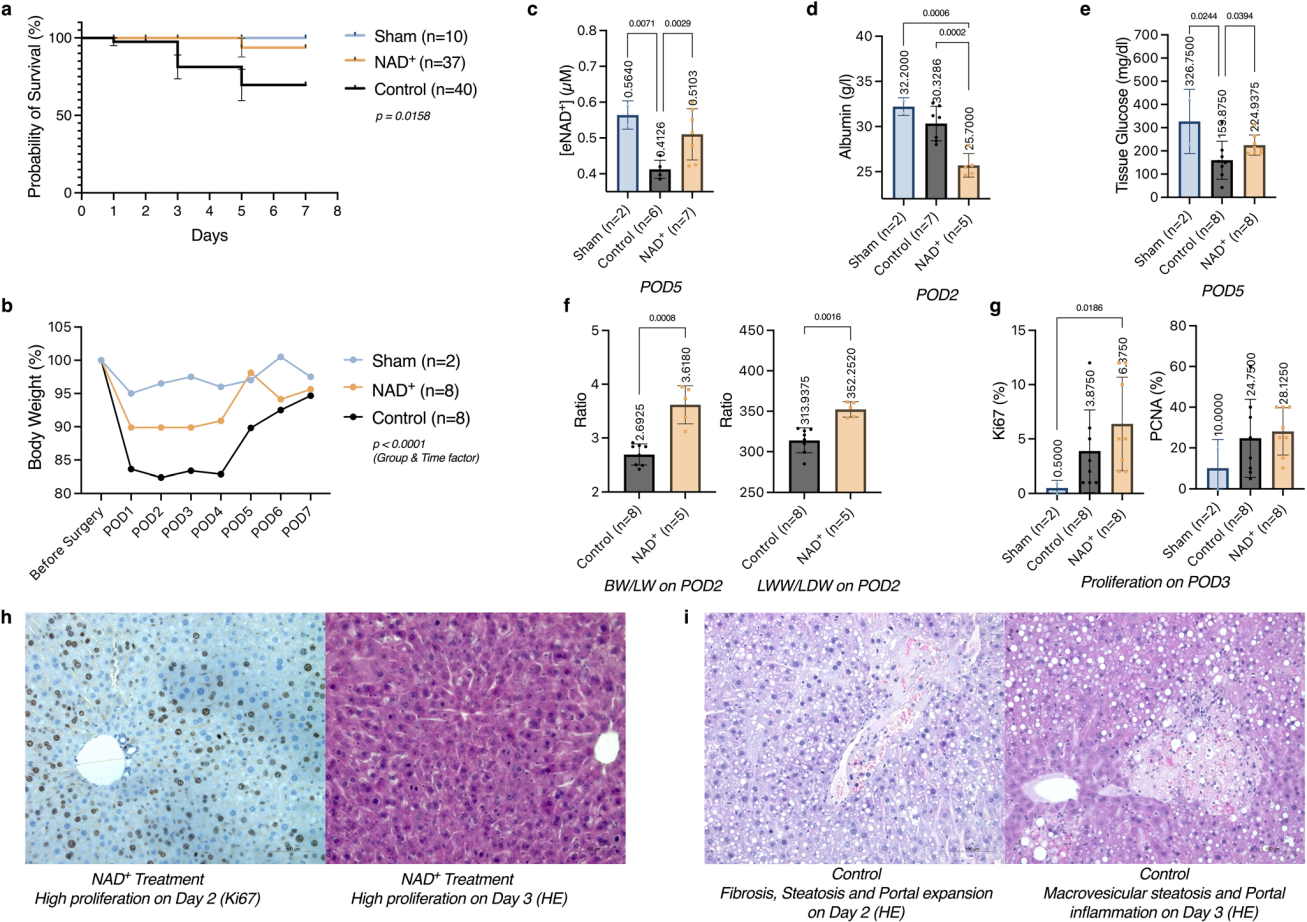
Extended liver resection in mice under NAD^+^ treatment. Wild type male *C57BL/6* mice of 8-10 weeks of age undergoing extended liver resection under 6 mg NAD^+^ (treatment) or saline (control) treatment versus sham surgery (laparotomy without resection). **a** Probability of survival compared with log-rank test for trend. **b** Post-operative weight deviation from baseline weight analysed with two-way ANOVA. **c** Plasma eNAD^+^ uptake of intraperitoneal NAD^+^ injections compared on post-operative day (POD) 5 with Kruskal-Wallis test. **d** Regenerative capacity of the liver following resection compared on POD 2 with the Mann-Whitney-U test. **e** Immunohistochemical markers for proliferation, Ki67 and PCNA, are displayed as percentages of proliferating cells on POD3 compared with Kruskal-Wallis test. **f** Blood albumin compared on POD2 and **g** glucose preservation in liver tissue on POD3, both analysed with Kruskal-Wallis test. **h** High cell proliferation rate and regeneration seen in Ki67 and H&E staining following extended liver resection under NAD^+^ treatment and post-operative fibrosis, steatosis; and **i** portal expansion with inflammation in the control group. Bars represent mean levels with scatter plot, error lines for standard deviation.

We evaluated NAD^+^ treatment in a murine model of extended liver resection, comparing NAD^+^ treated, saline-treated control, and non-resected sham groups. Cumulative analysis of these experiments revealed a significantly enhanced probability of survival after an extended liver resection with an intraperitoneal NAD^+^ treatment (*p* = 0.0158). The survival proportions on POD 7 were 69.6% for the control group, 93.8% for the NAD^+^ treatment and 100% for the sham group (Fig. 6a). Upon clinical monitoring of the mice, all three groups presented with instantaneous weight loss on the first post-operative day, with control mice having the highest weight drop, while this effect was mitigated in the NAD^+^ treatment group (Fig. 6b, *p* < 0.0001). The sham group showed the lowest deviation from the pre-operative baseline weight.

Additionally, we measured eNAD^+^ in plasma samples from the mice on POD5, to demonstrate the uptake of NAD^+^ from the intraperitoneal cavity into the bloodstream. NAD^+^ treated mice revealed elevated eNAD^+^ plasma levels (*n* = 7, *M* = 0.510 ± 0.071 µM, *p* = 0.0029) compared to the control group (*n* = 6, *M* = 0.413 ± 0.025 µM, Fig. 6c). Note, sham mice who did not undergo liver resection showed the statistically significantly highest plasma eNAD^+^ (*n* = 2, *M* = 0.564 ± 0.039 µM, *p* = 0.0071) among all groups, despite not receiving any NAD^+^ treatment. Albumin levels in blood were measured and appeared to be lowest in the NAD^+^ treatment group (POD2, *n* = 5, *M* = 25.70 ± 1.30 g/l) in contrast to both the control group (*n* = 7, *M* = 30.33 ± 1.90 g/l, *p* = 0.0002) and sham group (*n* = 2, *M* = 32.20 ± 0.99 g/l, *p* = 0.0006, Fig. 6d). In contrast, NAD^+^ treated mice exhibited a higher glucose retention in liver tissues (*n* = 8, *M* = 224.94 ± 43.49 mg/dl) than the control group (*n* = 8, *M* = 159.88 ± 82.07 mg/dl, *p* = 0.0394, Fig. 6e). The regenerative capacity of the liver tissue was assessed through body weight to liver weight (BW/LW) and liver wet weight to liver dry weight (WW/DW) ratios on the second post-operative day, where the highest regeneration rate was expected^26^. The NAD^+^ treatment group demonstrated higher ratios of BW/LW and WW/DW (*M* = 3.62 ± 0.35, *p* = 0.0008; *M* = 352.25 ± 9.43, *p* = 0.0016, ratios respectively), compared to the control group (*M* = 2.69 ± 0.20 and *M* = 313.94 ± 15.41, Fig. 6f). Following up on this finding, *Ki67* staining of liver tissues following liver resection exhibited higher proliferation in the NAD^+^ treatment group (*n* = 8, *M* = 6.38 ± 4.31%) versus the control group (*n* = 8, M = 3.88 ± 3.80%), when compared to the sham group (Fig. 6g). *PCNA* staining of the liver tissue revealed a similar observation, albeit without reaching statistical significance. Finally, the NAD^+^ treated mice exhibited enhanced cell proliferation and regeneration (Fig. 6h). Meanwhile, the representative histopathological slides with H&E staining also show increased fibrosis, steatosis, and portal expansion with inflammation in the control group (Fig. 6i).

## Discussion

In this study, we explore the importance of **extracellular NAD**^**+**^ in the context of liver fibrosis and present substantiation of its prospective regenerative characteristics, from bench to bedside. One of the most comprehensive and excellent reviews incorporating liver fibrosis and the NAD^+^ metabolome was published in 2022 by Dall et al.^27^. In the conclusion of this review, it is made clear that many aspects of the basic NAD^+^ metabolism still require further attention and more translational research. A crucial part of the basic NAD^+^ homeostasis, the eNAD^+^, had been left unrevealed due to technical shortcomings in the quantification. For the first time, we employed a previously described quantification method of eNAD^+^ in a cohort of liver surgery patients and aimed to translate eNAD^+^ levels in plasma into liver fibrosis progression and post-operative clinical course. We supported our concept of NAD^+^ induced hepatic regeneration and increased cell viability in vitro with hepatocyte cultures and in vivo with a mouse model of extended liver resection.

The careful assessment of the anticipated liver remnant´s volume and function prior to surgery is imperative to mitigate the potential occurrence of post-operative complications, including Post-Hepatectomy Liver Failure (PHLF) and determines surgical strategy and oncological radicality. This precautionary measure becomes necessary due to the presence of chronic liver diseases such as cirrhosis^28^. In our cohort of more than 90 resection patients, only a relatively small number of patients (12%) with higher fibrosis stages (F3 and F4) were found eligible for a major resection compared to the patients (75%) with lower fibrosis stages (F1 and F2) (Fig. 1a-c). The likelihood of successful liver resection diminishes progressively as liver function deteriorates, due to insufficient liver function and increased risk for post-operative complications. 16% of the liver resection patients in our cohort were either just before or during the surgical procedure found to be ineligible for the planned surgery due to contraindications, such as cirrhosis (33% of the aborted surgeries) or tumour progression or peritoneal carcinosis. Our findings allign with the literature both progressing liver fibrosis or cirrhosis and major resections independently resulted in reduced overall survival and higher risk of post-operative complications, along with reduced recurrence-free rates for cirrhosis patients^29–31^.

PHLF has a varying incidence of up to 35% and contributes significantly to mortality after liver resection^32^. Investigating the relevance of eNAD^+^ in liver function is crucial. In our cohort, we found an incidence of PHLF of 10.5% (10 out of 95 liver resection patients). In order to prevent PHLF, assessment of pre-operative liver function and fibrosis stage gains even more importance in planning liver resections^33,34^. The translational value of eNAD^+^ in this context depends on its alignment with the well-established parameters. Remarkably, eNAD^+^ levels were relatively upregulated in patients suffering from PHLF (Fig. 1d), although the causality behind this observation remains speculative. One of the key parameters in the ISGLS criteria^24^ for liver failure, bilirubin, revealed a positive correlation with the measured eNAD^+^ levels (Fig. 1e). We sought to determine whether the alterations in eNAD^+^ levels were associated with post-operative liver function and fibrosis, which is one of the main pre-defined causes of post-operative liver insufficiency^35^. We monitored the patients who underwent liver resection during their recovery and we found that eNAD^+^ levels decreased post-operatively. However, this effect diminished in patients with progressed liver fibrosis (especially for patients with F3/F4 fibrosis) (Fig. 2).

The liver not only consumes the most tryptophan for NAD^+^ biosynthesis^18^, but is also capable of excreting nicotinic acid (NA) and supplying other organs with NAD^+^ precursors via haemocirculation^17^. In their 2020 study, Parker et al. investigated the NAD^+^ metabolome of the human liver. Their findings revealed significantly lower levels of NAD^+^ and NAD^+^ precursors in patients suffering from alcohol-related liver disease (ALD) in comparison to other non-alcohol-related liver diseases. Furthermore, progressing ALD was linked to lower liver tissue NAD^+^ levels^36^. However, this data is about intracellular levels and does not cover the extracellular compartment. In conclusion, we observed that patients who suffered from liver insufficiency following liver resection exhibited elevated post-operative levels of eNAD^+^. Moreover, as liver fibrosis progressed and bilirubin levels increased, there was a corresponding increase in eNAD^+^ levels. Hence, it can be inferred that eNAD^+^ levels are upregulated with impaired liver function and fibrosis after liver surgery. In light of the findings regarding the upregulation of eNAD^+^ both in PHLF and higher stages of liver fibrosis suggest the role of eNAD^+^ as a systemic response as opposed to the intracellular concentrations reported in the literature.

Transaminase levels are known to increase following liver resection^37^, indicating cell death, including apoptosis and necrosis, which is why they are often employed as primary outcome measures in clinical trials^38^. At the same time, the release of NAD^+^ into the extracellular space can occur due to apoptosis or necrosis^39^. Following up on the extent of liver resection we compared levels of transaminases before and after surgery, considering the established value of transaminases as independent markers of hepatic tissue injury and post-operative complications. Our analyses of post-operative transaminase upsurges revealed significantly higher peak AST levels of patients undergoing major resections in contrast to patients with minor resection (Fig. 3a). However, major liver resections, naturally require longer operative times and bear higher risks for complications, which has been described in the context of post-operative transaminase levels acting as predictors of post-operative mortality and morbidity^40^. Unsurprisingly, operative time correlated with AST peaks post-operatively in our patient cohort (Fig. 3b). Analogously, Olthof et al. revealed in a study^37^ of more than 500 patients a positive correlation between operative time and the peak transaminases independently of the Pringle manoeuvre (clamping of the hepatoduodenal ligament to restrict hepatic vascular inflow). Eventually we managed to expose a positive correlation between eNAD^+^ and peak AST levels (Fig. 3c). Although this correlation was weak by numbers, it should be emphasised that there was a link between eNAD^+^ and AST on a systemic level. The literature describes a close association between AST and the maintenance of intracellular NAD^+^. This association is facilitated through the malate-aspartate shuttle, which plays a crucial role in glycolysis^41^ To our knowledge, this is the first reporting of eNAD^+^ in relation to liver injury following liver resection, through a correlation with transaminase, which is utilised religiously in the clinic to oversee post-operative complications. As opposed to the AST, peak levels of post-operative ALT did not exhibit a statistically significant correlation with the post-operative peak eNAD^+^ levels. AST presides predominantly in mitochondria, in contrast to ALT, that is found to a larger extent in cytoplasm. AST occurs to be less specific and sensitive for the liver, since it is not only present in liver cells but also in in other tissues, such as muscles. A further major difference between the two transaminases is the half-life in the circulation: while ALT is being cleared by sinusoidal cells in 47 h, only 17 hours are required for AST^42^. In light of these differences, an upsurge of AST greater than ALT may suggest greater cell injury, since the mitochondria are not damaged as easily as the cell membrane^43^. In fact, the mitochondria as the main residence and yielding the highest concentrations for both AST and NAD^+^ ^44^ serves as another explanation for the correlation we described. Please note, as a consequence of interindividual differences in eNAD^+^ metabolism, post-operative healing processes, and metachronous peak development of certain parameters, we decided to consider individual peak values in Figs. 1 and 3 of each parameter in our statistical analysis. Concisely, our observations indicate that liver fibrosis is a principal limiting factor for liver resection and, transaminase levels, as an indicator of cell death, correlate not only with the extent of the liver resection and operative time but also notably in the case of AST with post-operative peak eNAD^+^ levels.

NAD^+^ can be generated through various ways from endogenous and exogenous precursors, including nicotinamide, tryptophan, and nicotinamide riboside. Correspondingly, we quantified the expression of the rate limiting enzymes in the NAD^+^ salvage pathway, namely NAMPT and NMNAT3, in liver tissue through immunochemical staining. The trend of increasing eNAD^+^ in progressing liver fibrosis was supported by the increased tissue expression of these two rate-limiting enzymes in cirrhotic liver tissue of our patient cohort (Fig. 4). Furthermore, this finding sheds light on the source of eNAD^+^, which we suggested as a systemic response to the the onset liver dysfunction. We believe that the liver may utilise from its last remanining capacities self generated NAD^+^ to recruit further resources. With an approximately 46-fold lower enzymatic activity than NMNAT, NAMPT symbolises the rate-limiting enzyme in the NAD^+^ biosynthesis^45,46^ and is found both intra- and extracellularly^47,48^, while the global absence of this enzyme in mice is always lethal^49^. However, RNA expression of these enzymes did not show increased levels in cirrhotic patients, when we utilised an external dataset^25^ containing transcriptomic data of 216 samples collected from multiple centers by Govaere et al. (Fig. 4e, f). The discrepancy of genomic expression between RNA and protein levels is however, a phenomenon, which has been described in the literature before^50,51^ and it is substantiated by many levels of cellular regulation overriding the transcriptional level of proteins. In summary, the expressions of the rate-limiting enzymes for NAD^+^ biosynthesis, namely NAMPT and NMNAT3 were found to be upregulated on the protein level, through immunohistochemical stainings on our own specimens. However, the corresponding RNA expression of NAMPT and NMNAT3 in the liver tissue from external data did not yield a statistically significant difference.

In previous sections, we demonstrated that patients had lower eNAD^+^ levels after liver resection, while the patients with liver fibrosis and PHLF exhibited upregulated eNAD^+^ status. Therefore, we evaluated whether NAD^+^ treatment affects liver viability in a pre-clinical setting. The use of a 3D liver cell cultures, mimicing spheroid formation can help study the effects NAD^+^ metabolome on cellular inflammation and apoptosis, and supports the beneficial implementation of a NAD^+^ treatment. *TNFalpha*, which was employed in the in vitro model to emulate liver injury, has been previously described and discussed in its dual function^52^. (Fig. 5) Cell viability evaluation of *TNFalpha* and NAD^+^ treated hepatocytes in our experimental setup not only exhibited the hepatotoxic character of *TNFalpha* but also demonstrated the therapeutic potential of NAD^+^ in salvaging the aforementioned cell damage and apoptosis. One pitfall of the in vitro experiments was the baseline variation of viability measurements due to the manual cell counting method, yielding high deviation of initial number of cells in each treatment group. NAD^+^ precursors have been found to improve hepatic mitochondrial function and decrease oxidative stress in pre-clinical MASLD (previously known as *NAFLD*) models^27^. In conclusion, the in-vitro data demonstrates that treating organoids of primary murine hepatic cells with NAD^+^ significantly enhances the viability and might protect from *TNFalpha* induced cell-death.

In an experimental mice study^53^ of Sambeat et al. the absence of *NRK1*, a rate-limiting enzyme of NAD^+^ synthesis from a precursor, nicotinamide riboside (NR), resulted in a reduced gluconeogenic capacity and compromised hepatic mitochondrial function. When mice were subjected to a high-fat diet, mice lacking *NRK1* exhibited glucose intolerance, insulin resistance, and hepatosteatosis. Similarly Gariani et al. demonstrated the beneficial effect of NR administration in halting the MAFLD progression in a mouse model^54^. While these studies focused on the NAD^+^ supplementation on tissue levels, we aimed to explore the extracellular presence of NAD^+^. Eventually, we explored the significance of NAD^+^ treatment in a murine model of extended liver resection using different groups: NAD^+^ treatment group, control group (saline treatment), and sham group with no liver resection. Inducing liver regeneration for experimental setups via liver resection in a mouse model is broadly used and well described in the literature. We utilised an established protocol of extended liver resection in a mouse model as published before^55^, whether an intraperitoneal NAD^+^ treatment elevates plasma eNAD^+^ levels and consequently modifies the regeneration process. For the mouse model, we used intraperitoneal injections as the method of NAD^+^ application. Intraperitoneal injection is clearly superior to oral administration, since bioavailability experiments showed that NAD^+^, when ingested, is hydrolysed in the small intestine^56^. The literature^55^ suggests a varying post-operative mortality after extended liver resection in mice between 0% and 50%. Our results lie within this range for both control and NAD^+^ treatment groups (Fig. 6a). Highest post-operative mortality was seen in the control group with 30.6% due to insufficient recovery following the surgery. Yet the NAD^+^ treatment appears to have improved post-operative survival, weight gain, higher ratios of BW/LW and LW/DW ratios on the day of peak cell proliferation (post-operative day 2). Concordantly to the gradual increase and the normalisation of the post-operative weight, NAD^+^ treated mice exhibited higher glucose levels in liver tissues, indicating improved utilisation and uptake of nutrients, which were equally available in all groups. Moreover, we were able to show that eNAD^+^ in plasma increased after intraperitoneal treatment of NAD^+^ (Fig. 6c). This indicates that the injection leads to a successful uptake of the administered NAD^+^ into the bloodstream. One of the contradictable results are the blood albumin levels on the second post-operative day (Fig. 6d). This finding can be interpreted in two different ways: Either the NAD^+^ administration impairs the liver function in albumin synthesis or the elevated plasma eNAD^+^ levels via application of NAD^+^, or not NAD^+^ precursors as in many of the previous experiments, interact with the albumin in blood.

In summary, the administration of NAD^+^ treatment to mice who undergoing extended liver resections significantly enhanced their survival rates, stimulated cellular proliferation, and resulted in elevated tissue glucose levels in the liver. Histological examination revealed reduced steatosis in the NAD^+^- treated mice, indicative of less transient regeneration-associated steatosis (TRAS)^57^, which is typically initiated after major liver resection (Fig. 6h, i).

In conclusion, we show, that eNAD^+^ is upregulated in post-hepatectomy liver failure, and rate-limiting enzymes from NAD^+^ biosynthesis exhibited higher expressions under liver fibrosis. At the same time, the administration of NAD^+^ both in vivo and in vitro was shown to improve survival after extended liver resection in mice and cell viability in hepatocyte culture. While the search results shed light on the systemic circulation and tissue-specific presence of NAD^+^ and its precursors^58^, they fall short of exploring the specific pathways through which eNAD^+^ enters cells or evades degradation. Detailed discussions on these mechanistic details are notably absent in the available literature. In order to investigate the dynamics of eNAD^+^ in regards to its consumption by NADases and intracellular flux the framework of eNAD^+^ presence needs to be established quantitatively. These findings, illustrating the positive effects of eNAD^+^ homeostasis on liver regeneration in humans and, may serve as a potential target for future therapeutic approaches on improving eligibility for liver resection.

## Methods

### Human cohort study

#### General characteristics of the study

The observational study was approved by the Charité ethics committee (Ethikkommission der Charité - Universitätsmedizin Berlin) under the vote number EA1/291/16 and EA1/018/17. It was registered on the German Clinical Trials Register (DRKS00012260) and was conducted in compliance with the local regulatory guidelines, the Declaration of Helsinki and the STROBE cohort reporting guidelines^59^. Patient recruitment took place between December 2016 and May 2018, with the following eligibility criteria: age ≥ 18, male or female, indication for liver resection, healthy control with no liver, neoplastic, psychiatric or severe internal medicine disease, obtained informed consent, ability to give consent. Patients who did not meet the eligibility criteria were excluded. 119 patients were recruited, among them 95 underwent liver resection and 24 underwent hernial repeair. The surgical approach and technique used was previously described by our department^60,61^. We divided the patient collective following liver resection into subgroups based on liver fibrosis, according to the Desmet score, tumour entity, operative time, NAMPT & NMNAT3 expression, and the incidence of PHLF. PHLF was classified according to the International Study Group of Liver Surgery (ISGLS) grading^24^ based on post-operative INR and bilirubin levels.

#### Blood sample collection

Patients who met the aforementioned inclusion criteria, were selected, informed about the study and recruited after giving informed consent. Peripheral venous fasted blood was collected into lithium heparin tubes once prior to surgery and again, until discharge, on post-operative days (POD) 1, 2, 5 and 10 for liver resection patients and on POD 1 and 2 for control patients. Due to the lack of exhaustive data on the pharmacological half-life of eNAD^+^, the samples were expeditiously centrifuged at 2500 g at 4 **°**C for 15 min. The separated plasma was aliquoted, snap frozen in liquid nitrogen and stored at −80 **°**C for later eNAD^+^ measurement.

#### Clinical parameters

All patients were evaluated in line with standard clinical management protocols for their respective disease, including routine blood tests, such as liver function tests including alanine transaminase (ALT), aspartate transaminase (AST), prothrombin time (PT) with international normalized ratio (INR), albumin, bilirubin and arterial blood gas analyses.

#### Histology & immunohistochemistry

Resected liver tissues from patients were examined histopathologically by our internal Institute of Pathology, in order to assess the specimens’ entity, malignancy, stage of fibrosis and fatty degeneration (steatosis). Collected liver specimens from mice were fixated in formaldehyde, embedded in paraffin, and subsequently sectioned in 4 µm and 2 µm thickness, for Meyers’ hematoxylin-eosin (H&E), *Ki67* and proliferating cell nuclear antigen (*PCNA*) staining. For the assessment of liver cell morphology, steatosis and fibrosis^62^, a standard protocol was used based on H&E staining. Meanwhile, the quantification of liver cell proliferation and regeneration was made utilizing *Ki67* and *PCNA* staining. Mayer’s hematoxylin was finally used as counterstaining. Micrographs were shot with a Zeiss Axio Observer Z1 microscope equipped with an AxioCam 1Cc5 for brightfield and fluorescence microscopy. Image analysis was done by Zen 2.3 Pro software (*Carl Zeiss AG, Oberkochen, Germany*). Staining estimation and cell counting were assisted by two independent pathologists from different institutions and the non-profit TMARKER software (*Thomas Fuchs Lab, Medical Machine Learning & Computational Pathology, New York, NY, USA*).

#### NAMPT/NMNAT staining in liver tissue

In addition to the standard procedure, we quantified the expression of both rate-limiting enzymes of the NAD^+^ salvage pathway, NAMPT and NMNAT3, in the liver resectates. Out of formalin-fixed, paraffin-embedded (FFPE) liver samples from 71 patients, who underwent liver resection, tissue-microarrays (TMA) were engineered and analysed histomorphologically, as described before^63^. Primary *anti-NAMPT* and *anti-NMNAT3* antibodies (brown) (*Thermo Fisher Scientific, Massachusetts, USA, Cat. MA5-43719 and PA5-63436, respectively*) were used in immunohistochemistry analyses using a streptavidin peroxidase conjugated method. To enhance the background contrast, the slides were counterstained with hematoxylin (blue). Expression was evaluated as an immunoreactivity score (IRS, from 0–12, 0–1 = negative, 2–3 = low, 4–8 = high, 9–12 = very high)^64^, calculated as the product of the percentage of stained cells (0 = 0%, 1 = 1-25%, 2 = 26-50%, 3 = 51-75%, 4 = 76-100%) and the staining intensity (score 0 = no color reaction to 3 =  intense reaction).

### Transcriptomic data

#### *NAFLD* patient cohort and RNA sequencing

Under the Gene Expression Omnibus accession number GSE135251, Govaere et al. created a library from a multicenter study of 216 snap frozen liver biopsies consisting of 206 *NAFLD* patients with a full range of normal liver tissue to cirrhosis and 10 control participants, which underwent high-throughput RNA sequencing^25^. We leveraged the processed data with expression values of two specific genes, namely NAMPT and NMNAT3, which were described earlier in detail as the rate-limiting enzymes of the NAD^+^ metabolome.

### 3D primary hepatocyte culture

#### Hepatocyte isolation

Following a careful cannulation and perfusion first with 20 mL of pre-warmed (37 °C) Leffert’s buffer enriched with 0.2 M EGTA (50 mL 1x Leffert’s + 1.25 mL of 0.2 M EGTA) at a flow rate of 1.6 mL/min then 20 mL of collagenase buffer (50 mL 1x Leffert’s + 0.5 mL 1 M CaCl_2_ + 50 mg collagenase) at a flow rate of 1.6 mL/min of the liver in C57BL/6/N mice, the explanted organ was mechanically minced and washed in a vessel containing a BSA buffer (50 mL 1x Leffert’s + 1 mL 1 M CaCl_2_ + 3 g BSA) on ice. Sterile filtered cells were then counted with a hemocytometer and on day -7, 2000 hepatocytes/well were added to a 96-well ultra-low-attachment (ULA) plate (*CellCarrier Spheroid ULA 96-well Microplates, PerkinElmer, USA, Cat. 6055330*). The peripheral wells were filled with medium only to avoid evaporation-induced damage. This was followed by a centrifugation at 100 x g for 2 minutes and finally incubation at 37 °C and 5% CO_2_.

#### Hepatocyte culture and viability experiments

Medium change with spheroid medium without fetal bovine serum (FBS) was done on days -5, -4 and -3. The plates were left undisturbed in the incubator during the spheroid formation. Starting with day -2, a medium change took place every 48 h. The spheroid formation process took 7 days. Thus, experimental proceedings began on Day 0. The beginning of the experiment and the progress on day 10 were captured via immunofluorescence staining and microscopy, following cryopreservation. Cytokeratin 18 (*Anti-Cytokeratin 18 Antibody (APC-Cy5.5), Abcore, Cat. AC12-0095-04*) with DAPI (*1* *mg/mL, ThermoFisher Scientific, Cat. 62248*) counterstaining and albumin (*594-conjugated Albumin Polyclonal antibody, CoraLite, Cat. CL594-16475*) were utilised to visualise hepatocytes. All spheroids were distributed into 4 groups on day 0: *Control* (no *TNFalpha* & no NAD^+^), *Treatment 1* (100 ng/mL *TNFalpha*, *abcam, Cat. ab9642*), *Treatment 2* (500 ng/mL NAD^+^, *Sigma-Aldrich, Cat. N3014*), *Treatment 3* (100 ng/mL *TNFalpha* & 500 ng/mL NAD^+^). As a hepatotoxic compound, the *TNFalpha* treatment emulated liver tissue insults (such as surgical tissue trauma) by inducing acute liver cell inflammation and triggered cell death. Cell viability was assessed on days 0, 2, 6 and 10, via the CellTiter-Glo Luminescent Cell Viability Assay (*Promega, Cat. G7570*).

Further details on all methods used are provided in the Supplementary Data 1.

### Animal experiments

#### Animals, housing and NAD^+^ treatment

All animal procedures were approved (G0205/15) by the Regional Office for Health and Social Affairs Berlin (Landesamt für Gesundheit und Soziales, LaGeSo Berlin) for animal welfare and testing in accordance with the European directive 2010/63/EU of the European Parliament and of the Council for the protection of animals used for scientific purposes and ARRIVE1 guidelines^65^. For the experiments, wild type male *C57BL/6/N* mice (*n* = 90) of 8–10 weeks of age were acquired from the Research Facilities for Experimental Medicine (Forschungseinrichtungen für Experimentelle Medizin, FEM) facilities of Charité Universitätsmedizin Berlin. These were housed in groups of 4–5 mice per cage on a 12 h light-dark cycle with ad libitum access to food and water. Animals were randomly assigned to 3 groups: treatment, control, sham and were observed for different lengths of time following the undermentioned extended liver resection: 1, 2, 3, 5 and 7 days. Daily intraperitoneal injections of 6 mg NAD^+^ (*Sigma-Aldrich, Cat. N3014)* dissolved in 100 µL PBS were given to mice in the treatment group while PBS was administered to the control group mice, starting 3 days prior to surgery and continuing daily during the post-operative course until termination. Sham mice did not receive any treatment. All groups were observed for 1, 2, 3, 5, and 7 days until finalisation.

#### Extended liver resection and sham procedure in mice

Male wild-type *C57BL/6 N* mice, randomly assigned in control and NAD^+^ treatment groups, underwent an extended liver resection based on the protocol^55^ from Kamali et al. in order to induce liver regeneration. Following subcutaneous analgo-sedation with ketamine/xylazine and inhalative isoflurane anaesthesia, a frontal midline incision was carried out. Once the medial and left lateral lobes were presented, these were ligated and resected thereafter. Blood glucose in liver tissue was measured by glucometer (*FreeStyle Precision Neo, Abbott Diabetes Care, Cat. 7214052*). After ruling out residual bleeding, the abdominal cavity was closed by separate suturing of the peritoneum and cutaneous tissue. The sham procedure consisted only of a laparotomy without performing any liver resection. The mice received metamizole and a post-operative observation in a heated chamber.

#### Post-operative assessment

During the pre- and post-operative observational phase each mouse was weighed and assessed for their health status. Moribound animals were registered, excluded from the experiment and euthanized immediately. Animals were sacrificed on post-operative days 1, 2, 3, 5 and 7, as indicated above. Regenerating liver remnant and blood samples were collected after finalisation. Blood glucose in the remnant liver tissue was measured via glucometer as above. Peripheral liver tissue and centrifuged blood plasma were snap-frozen in liquid nitrogen. Another part of the liver tissue was collected and stored in formaldehyde for histological/immunohistochemical assessment. All remaining liver tissue was dried to assess the ratio of dried liver weight to wet liver weight.

### Extracellular NAD^+^ quantification

eNAD^+^ concentrations in both human and mouse plasma, were quantified according to a novel enzymatic two-step cycling assay, which was established in our research group and described in a previous publication^23^ by Brunnbauer et al. In order to define eNAD^+^ concentrations within samples, a set of β-NAD^+^ (β-Nicotinamide-adenine-dinucleotide hydrate, Sigma-Aldrich, Cat. N3014) standards, with predefined concentrations in a 6 step 2-fold serial dilution, were used to construct a standard calibration curve through the origin (S1 to S6 0.7536 µM to 0.0235 µM, respectively), with the dependent variable being the rate of change of absorbance (or rather the speed of the reaction). In order to assure the comparability and reproducibility of the measurements, each plate included 2 sets of quality controls (QC1 to QC6) with the same predefined concentrations as the standards.

### Statistics and reproducibility

All data are available from the corresponding author on reasonable request. Quantitative data were analysed using descriptive and inferential statistics. Descriptive statistics, including means (*M*), mean differences (*MD*), standard deviations, and percentages, were calculated to summarise the demographic characteristics and clinical variables. Prior to initiating any analyses, the data were cleaned and assessed for normality using the Shapiro-Wilk test. To compare differences between groups, parametric tests for normally distributed data, such as ANOVA, as well as non-parametric tests, such as Mann-Whitney U and Kruskal-Wallis tests, were utilised. Contingency tables were analysed with Chi-squared tests. Repeated measures ANOVA is not equipped to handle missing values in our data. To overcome this limitation, we employed an alternative approach by utilising a mixed model analysis, which utilises a compound symmetry covariance matrix and is fitted using Restricted Maximum Likelihood (REML). In cases of no missing values, this methodology yields identical P-values (*p*) and multiple comparisons tests as those obtained from repeated measures ANOVA. However, in situations where missing values exist and adhere to the assumption of missing completely at random, the results can be interpreted similarly to those obtained from repeated measures ANOVA. Pearson’s or Spearman’s rank correlation coefficients were computed to evaluate associations between continuous variables, depending on their normality assessment. All statistical tests were two-sided, and the significance level was set to 5%. For statistical analysis, Graphpad’s Prism 9 (GraphPad Software, La Jolla, CA, USA) was used.

Despite efforts to minimise data loss due to follow-ups during the study, by maintaining regular contact with patients in the clinical trial, some loss to follow-up was inevitable, primarily due to variations in hospitalisation duration following surgery. A complete case analysis (CCA) was employed, which included only those participants with complete data for all variables of interest. This straightforward approach enabled us to analyse the relationships between variables without imputing or estimating any missing values. The potential impact of the missing data on our results was assessed by comparing the characteristics of participants with complete data to those with missing data. This comparison aimed to identify any systematic differences between the two groups, which could potentially introduce bias into our findings.

### Reporting summary

Further information on research design is available in the Nature Portfolio Reporting Summary linked to this article.

### Supplementary information

Description of Additional Supplementary Files

Supplementary Data 1

Supplementary Data 2

Reporting summary

## Data Availability

The datasets generated during and/or analysed during the human, animal and cell culture studies are available in Supplementary Data 2. The transcriptomic data that support the findings of this study are available in the Gene Expression Omnibus with the identifier GSE135251^25^.
