# Supplementary material for: Extracellular NAD+ response to post-hepatectomy liver failure: bridging preclinical and clinical findings
Source: Commun Biol. 2024 Aug 14;7:991. doi: 10.1038/s42003-024-06661-0 (PMC11324947; doi:10.1038/s42003-024-06661-0)
Supplement: Supplementary file 1 — Description of Additional Supplementary Files [file 42003_2024_6661_MOESM1_ESM.pdf]

Description of Additional Supplementary Files

File name: Supplementary Data 1
Description: CTAT methods table

File name: Supplementary Data 2
Description: The datasets generated during and/or analyzed during the human, animal and cell culture studies
