# Supplementary material for: Extracellular NAD+ response to post-hepatectomy liver failure: bridging preclinical and clinical findings
Source: Commun Biol. 2024 Aug 14;7:991. doi: 10.1038/s42003-024-06661-0 (PMC11324947; doi:10.1038/s42003-024-06661-0)
Supplement: Supplementary file 2 — Supplementary Data 1 [file 42003_2024_6661_MOESM2_ESM.pdf]

CTAT methods table

1.1 Antibodies

**Name**	**Citation**	**Supplier**	**Cat no.**	**Clone no.**
Ki-67 Polyclonal Antibody		Invitrogen	PA5-19462	
Proliferating Cell Nuclear Antigen		DAKO	M0879	
NAMPT Monoclonal Antibody		Invitrogen	MA5-43719	
NMNAT3 Polyclonal Antibody		Invitrogen	PA5-63436	
Anti-Cytokeratin 18 Antibody (APC-Cy5.5)		Abcore	AC12-0095-04	
94-conjugated Albumin Polyclonal antibody		CoraLite	CL594-16475	
DAPI		ThermoFisher Scientific	62248	
100 ng/mL TNFalpha		abcam	ab9642	

1.2 Cell lines

**Name**	**Citation**	**Supplier**	**Cat no.**	**Passage no.**	**Authentication test method**

1.3 Organisms

**Name**	**Citation**	**Supplier**	**Strain**	**Sex**	**Age**	**Overall n number**
Black 6		Forschungseinrichtungen für Experimentelle Medizin, FEM – Charité	C57BL/6	m	8-10 weeks	

1.4 Sequence based reagents

Name	Sequence	Supplier

1.5 Biological samples

Description	Source	Identifier

1.6 Deposited data

Name of repository	Identifier	Link
Gene Expression Omnibus	GSE135251	https://www.ncbi.nlm.nih.gov/geo/query/acc.cgi?acc=GSE135251

1.7 Software

Software name	Manufacturer	Version
Prism	GraphPad Software, LLC	Version 9.3.1
TMARKER	Thomas Fuchs Lab, Medical Machine Learning & Computational Pathology, New York, NY, USA	
Zen Pro	Carl Zeiss AG	Version 2.3

1.8 Other (e.g. drugs, proteins, vectors etc.)

β-Nicotinamid-adenin-dinucleotid Hydrat	Sigma-Aldrich	N3014
Alcohol-Dehydrogenase from Saccharomyces cerevisiae	Sigma-Aldrich	A7011
Thiazolyl Blue Tetrazolium Bromide	Sigma-Aldrich	M2128
Phenazine methosulfate	Sigma-Aldrich	P9625
Triethanolamine	Sigma-Aldrich	90279
Triethanolaminhydrochlorid, +99 %	Thermo Scientific Chemicals	170051000
Bovine Serum Albumin	Sigma-Aldrich	A7030
Protein Block-Serum-Free	DAKO	X0909
REAL Peroxidase-Blocking-Solution	DAKO	S202386
Streptavidin-HRP	DAKO	P0397
LSAB2 System, HRP Biotinylated Link	DAKO	K0672
CellTiter-Glo Luminescent Cell Viability Assay	Promega	G7570
CellCarrier Spheroid ULA 96-well Microplates	PerkinElmer, USA	6055330
Hepatocyte Wash Medium	Gibco, ThermoFisher Scientific	17704024
Zeiss Axio Observer Z1	Carl Zeiss AG, Oberkochen, Germany	
AxioCam 1Cc5	Carl Zeiss AG, Oberkochen, Germany	
Infinite 200 PRO	Tecan, Switzerland	
FreeStyle Precision Neo	Abbott Diabetes Care	7214052
