# Supplementary material for: Extracellular NAD+ response to post-hepatectomy liver failure: bridging preclinical and clinical findings
Source: Commun Biol. 2024 Aug 14;7:991. doi: 10.1038/s42003-024-06661-0 (PMC11324947; doi:10.1038/s42003-024-06661-0)
Supplement: Supplementary file 4 — Reporting summary [file 42003_2024_6661_MOESM4_ESM.pdf]

nature portfolio

Corresponding author(s): COMMSBIO-23-5115-T

Last updated by author(s): Jul 30, 2024

Reporting Summary

Nature Portfolio wishes to improve the reproducibility of the work that we publish. This form provides structure for consistency and transparency in reporting. For further information on Nature Portfolio policies, see our Editorial Policies and the Editorial Policy Checklist.

Statistics

For all statistical analyses, confirm that the following items are present in the figure legend, table legend, main text, or Methods section.

n/a	Confirmed	
☐	☒	The exact sample size (*n*) for each experimental group/condition, given as a discrete number and unit of measurement
☐	☒	A statement on whether measurements were taken from distinct samples or whether the same sample was measured repeatedly
☐	☒	The statistical test(s) used AND whether they are one- or two-sided<br>*Only common tests should be described solely by name; describe more complex techniques in the Methods section.*
☐	☒	A description of all covariates tested
☐	☒	A description of any assumptions or corrections, such as tests of normality and adjustment for multiple comparisons
☐	☒	A full description of the statistical parameters including central tendency (e.g. means) or other basic estimates (e.g. regression coefficient) AND variation (e.g. standard deviation) or associated estimates of uncertainty (e.g. confidence intervals)
☐	☒	For null hypothesis testing, the test statistic (e.g. F, t, r) with confidence intervals, effect sizes, degrees of freedom and P value noted<br>*Give P values as exact values whenever suitable.*
☒	☐	For Bayesian analysis, information on the choice of priors and Markov chain Monte Carlo settings
☐	☒	For hierarchical and complex designs, identification of the appropriate level for tests and full reporting of outcomes
☐	☒	Estimates of effect sizes (e.g. Cohen's *d*, Pearson's *r*), indicating how they were calculated

Our web collection on statistics for biologists contains articles on many of the points above.

Software and code

Policy information about availability of computer code

Data collection	No software used.
Data analysis	Graphpad's Prism 9 (GraphPad Software, La Jolla, CA, USA)

For manuscripts utilizing custom algorithms or software that are central to the research but not yet described in published literature, software must be made available to editors and reviewers. We strongly encourage code deposition in a community repository (e.g. GitHub). See the Nature Portfolio guidelines for submitting code & software for further information.

Data

Policy information about availability of data

All manuscripts must include a data availability statement. This statement should provide the following information, where applicable:

- Accession codes, unique identifiers, or web links for publicly available datasets
- A description of any restrictions on data availability
- For clinical datasets or third party data, please ensure that the statement adheres to our policy

The datasets generated during and/or analysed during the human, animal and cell culture studies are available in Supplementary Data 2.
The transcriptomic data that support the findings of this study are available in the Gene Expression Omnibus with the identifier GSE135251

Research involving human participants, their data, or biological material

Policy information about studies with human participants or human data. See also policy information about sex, gender (identity/presentation), and sexual orientation and race, ethnicity and racism.

Reporting on sex and gender	In our clinical trial, sex and gender-based studies were not conducted, as the main focus of the research was on the role of extracellular NAD+ in liver fibrosis and post-hepatectomy liver failure, rather than on sex or gender differences. Therefore, disaggregated sex and gender data were not collected or analyzed. Details of the study's focus and design can be found in the attached manuscript.
Reporting on race, ethnicity, or other socially relevant groupings	In our manuscript, we did not use socially constructed or socially relevant categorization variables such as race, ethnicity, or socioeconomic status, as the primary focus of our study was on the biochemical and clinical aspects of extracellular NAD+ in liver fibrosis and post-hepatectomy liver failure. Therefore, no such variables were included or analyzed in our research.<br><br>We defined and classified our study participants based on clinical and biological criteria relevant to the study's objectives, such as liver fibrosis stage, extent of liver resection, and levels of extracellular NAD+. These classifications were provided by the researchers based on clinical assessments and laboratory measurements, not self-report or administrative data.
Population characteristics	The covariate-relevant population characteristics of the human research participants in our study included age, liver fibrosis stage, and clinical diagnosis. The participants ranged in age from 26 to 86 years. The cohort included patients with various stages of liver fibrosis and different diagnoses, such as hepatocellular carcinoma, intrahepatic cholangiocellular carcinoma, and metastasis of colorectal cancer, among others. Detailed demographic and clinical characteristics, including past and current diagnoses and treatment categories, are provided in the manuscript's Methods section.
Recruitment	Participants were recruited from patients scheduled for liver resection at Charité – Universitätsmedizin Berlin. Eligible patients were identified based on criteria such as age ≥18 years, indication for liver resection, and absence of severe internal medicine or psychiatric diseases. Informed consent was obtained from all participants. Potential self-selection bias may be present, as patients who consented to participate might have different health conditions or motivations compared to those who declined. This bias could impact the generalizability of the results, as the study population may not fully represent the broader patient population undergoing liver resection.
Ethics oversight	The study protocol was approved by the Charité ethics committee (Ethikkommission der Charité Universitätsmedizin Berlin) under the vote numbers EA1/291/16 and EA1/018/17.

Note that full information on the approval of the study protocol must also be provided in the manuscript.

Field-specific reporting

Please select the one below that is the best fit for your research. If you are not sure, read the appropriate sections before making your selection.

☒ Life sciences ☐ Behavioural & social sciences ☐ Ecological, evolutionary & environmental sciences

For a reference copy of the document with all sections, see nature.com/documents/nr-reporting-summary-flat.pdf

Life sciences study design

All studies must disclose on these points even when the disclosure is negative.

Sample size	The intended investigations of the samples are carried out in the laboratory under experimental conditions and a case number planning was not carried out a priori, as the possible differences between the parametric and non-parametric measured values to be measured are unknown.
Data exclusions	No data were excluded from the analysis.
Replication	All atempts at replication were succesful.
Randomization	The clinical trial conducted in our research did not involve randomization or blinding due to its nature as a prospective observational cohort study.
Blinding	The clinical trial conducted in our research did not involve randomization or blinding due to its nature as a prospective observational cohort study.

Reporting for specific materials, systems and methods

We require information from authors about some types of materials, experimental systems and methods used in many studies. Here, indicate whether each material, system or method listed is relevant to your study. If you are not sure if a list item applies to your research, read the appropriate section before selecting a response.

Materials & experimental systems

n/a	Involved in the study
☐	☒ Antibodies
☒	☐ Eukaryotic cell lines
☒	☐ Palaeontology and archaeology
☐	☒ Animals and other organisms
☐	☒ Clinical data
☒	☐ Dual use research of concern
☒	☐ Plants

Methods

n/a	Involved in the study
☒	☐ ChIP-seq
☒	☐ Flow cytometry
☒	☐ MRI-based neuroimaging

Antibodies

Antibodies used	(For details please refer to the Supplementary data 1: PA5-19462, M0879, MA5-43719, PA5-63436, AC12-0095-04, CL594-16475, 62248, ab9642)
Validation	Detailed in the Methods section, including supplier names, catalog numbers, and validation information.

Animals and other research organisms

Policy information about studies involving animals; ARRIVE guidelines recommended for reporting animal research, and Sex and Gender in Research

Laboratory animals	For the experiments, wild type male C57BL/6/N mice (n = 90) of 8-10 weeks of age were acquired from the Research Facilities for Experimental Medicine (Forschungseinrichtungen für Experimentelle Medizin, FEM) facilities of Charité Universitätsmedizin Berlin.
Wild animals	The study did not involve wild animals.
Reporting on sex	Male mice were in the experiments to reduce variability from hormonal fluctuations, maintain consistency, utilize historical data, minimize physiological differences, and simplify logistical considerations.
Field-collected samples	The study does not involve field-collected samples.
Ethics oversight	All animal procedures were approved (G0205/15) by the Regional Office for Health and Social Affairs Berlin (Landesamt für Gesundheit und Soziales, LaGeSo Berlin) for animal welfare and testing in accordance with the European directive 2010/63/EU of the European Parliament and of the Council for the protection of animals used for scientific purposes and ARRIVE1 guidelines.

Note that full information on the approval of the study protocol must also be provided in the manuscript.

Clinical data

Policy information about clinical studies
All manuscripts should comply with the ICMJE guidelines for publication of clinical research and a completed CONSORT checklist must be included with all submissions.

Clinical trial registration	Registered on the German Clinical Trials Register (DRKS00012260)
Study protocol	The full trial protocol can be accessed via the German Clinical Trials Register (DRKS00012260). If further details are needed, please contact the corresponding author, Dr. Felix Krenzien, at felix.krenzien@charite.de.
Data collection	Data were collected at Charité – Universitätsmedizin Berlin between December 2016 and May 2018. Patients who met the eligibility criteria were recruited and provided informed consent. Peripheral venous blood samples were collected preoperatively and on postoperative days 1, 2, 5, and 10. Clinical parameters such as liver function tests, including alanine transaminase (ALT), aspartate transaminase (AST), prothrombin time (PT), albumin, bilirubin, and arterial blood gas analyses, were recorded following standard clinical management protocols.
Outcomes	The primary outcome was the level of extracellular NAD+ (eNAD+) and its correlation with post-hepatectomy liver failure (PHLF) and liver fibrosis stages. Secondary outcomes included the expression of NAD+ biosynthesis enzymes (NAMPT and NMNAT3). Outcomes were assessed using a combination of clinical measurements, laboratory assays, histopathological analysis, and statistical correlation methods detailed in the Methods section of the manuscript.

Plants

Seed stocks	No plants were used in this study.
Novel plant genotypes	Not applicable as no plants were involved in this study.
Authentication	Not applicable as no plants or novel plant genotypes were used in this study.
